# Dermatomyositis as the first manifestation of gallbladder adenocarcinoma: case report and literature overview

**DOI:** 10.1186/s12957-015-0535-4

**Published:** 2015-03-27

**Authors:** Petra Jurcic

**Affiliations:** Department of Radiation Therapy and Internal Oncology, Tumor Clinic, Sisters of Charity Hospital, Vinogradska 29, 10000 Zagreb, Croatia

**Keywords:** Dermatomyositis, Gallbladder, Adenocarcinoma, Paraneoplastic

## Abstract

Dermatomyositis (DM) is characterized by pathognomic cutaneous manifestations (heliotrope rash, periorbital edema, Gottron’s papules) and proximal muscle weakness. In this paper, I will present the case of a 48-year-old female patient whose dermatomyositis was initially diagnosed as vasculitis. Following the patient’s inadequate response to corticosteroid treatment, clinical and radiologic examinations were performed, showing inoperable gallbladder adenocarcinoma. Although initial chemotherapy led to regression, the dermatomyositis developed an independent course with new pathological changes leading to the progression of the disease. I will also present an overview of case reports in English published so far. Gallbladder carcinoma should be added to the list of malignancies with dermatomyositis and has to be excluded by relevant investigation in women.

## Background

Dermatomyositis (DM) is an uncommon inflammatory myopathy characterized by pain and weakness in the proximal muscles and cutaneous manifestations. The skin findings of DM include scaly violaceous papules and plaques overlaying the bony prominences of the hands (Gottron’s papules), violaceous patches on the periorbital skin (heliotrope eruption), photodistributed poikilodermatous patches and plaques, scaly plaques on the scalp and lateral thighs, periungual telangiectasia, and ragged cuticles [1]. Malignancies may occur before, simultaneously with or after the onset of DM [2]. The most common malignancies associated with DM are ovarian and lung cancer [3]. The pathogenesis of paraneoplastic DM is still relatively unknown, although there is some evidence to support the involvement of humoral and cell-mediated immune systems and the presence of tumour antigens provoking an autoimmune response [3]. Treatment of DM is empirical, often involving a combination of steroids and immunomodulatory drugs [4]. Where DM is associated with underlying malignancy, tumour therapy may lead to the resolution of DM. Interestingly, a reverse relationship also exists, whereby a flare of DM occurs with tumour recurrence - an association that is demonstrated in this report [5].

## Case presentation

**Figure 1:**
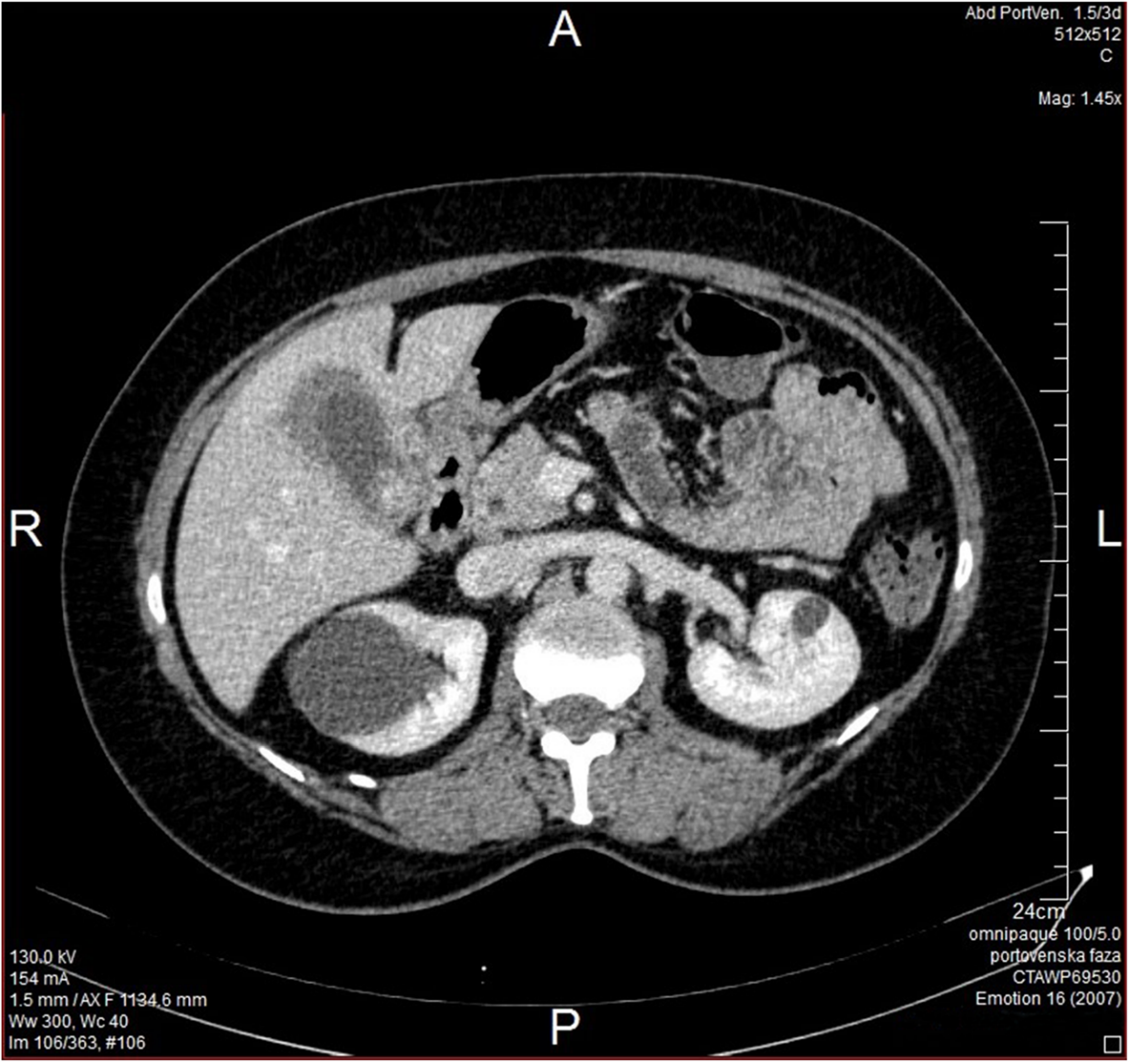
**Preoperative MSCT of the abdomen and the pelvis.**

A 48-year-old female patient with history of skull fracture suffered in a traffic accident 25 years ago presented in mid-January 2014 in the infectology clinic of her local hospital for facial, neck and ear erythema; butterfly-shaped rash; pain in the upper arm muscles; stiffness of hands and dysphagia. Considering the sedimentation (SE) 30 creatine kinase (CK) 308 result, as well as leukocyte (L) 11.4 (white blood cells (WBC) differential percent neutrophils (% neu) 87, percent lymphocytes (% lymphs) 7.2, percent monocytes (% monos) 5.9, percent eosinophils (% eos) 0), the patient was referred to immunology tests (anti-antinuclear antibodies verified by indirect immunofluorescence (ANA IIF) slightly positive and anti-double-strained DNA (anti-dsDNA), anti-Ro/SSA antibodies (anti-SS-A52), anti-Ro/SSA antibodies (anti-SS-A60), SSB/La antibody (anti-SSB), anti-Sm antibodies directed against 7 proteins (anti-Sm), autoantibodies directed against the RNP/Sm ribonucleoprotein complex (anti-Sm/RNP), anti-DNA topoisomerase 1 antibody (anti-DNA-topo 1), antibody directed against Jo-1 protein (anti-Jo-1), CENP-B specific anti-centromere autoantibodies (anti-CENP-B), autoantibodies to ribosomal P (anti-RIBO P) negative). Since the administered corticosteroid treatment yielded no improvement, a multi-slice computed tomography (MSCT) of the abdomen (Figure 1) was performed, showing a pronounced thickening of the cholecyst wall ranging from 11 to 17 millimeters (mm), scattered and eccentric in appearance; two intraluminally present choleliths, one within the fundus 18 × 16 mm and the other infundibular 22 × 16 mm; and no evident pericholecystic edema. The tumour markers are carcinoembryonic antigen (CEA) 0.7 μg/l and carbohydrate antigen 19-9 (CA 19-9) 964 U/ml. Laparoscopically visualized gallbladder showed malign alterations. Urgent cytological analysis showed malign cells, after which the procedure was reverted to laparotomy which verified omentum carcinomatosis, as well as infiltration of the fifth liver segment. Palpable lymph nodes were in the direction of the hepatoduodenal ligament. The procedure was finished with exploration. Final histopathology report of the omentum was adenocarcinoma (diffuse solid clusters of polymorphous atypical hyperchromatic tumour cells and gland formations with atypical mucous-cylindrical epithelium).

**Figure 2:**
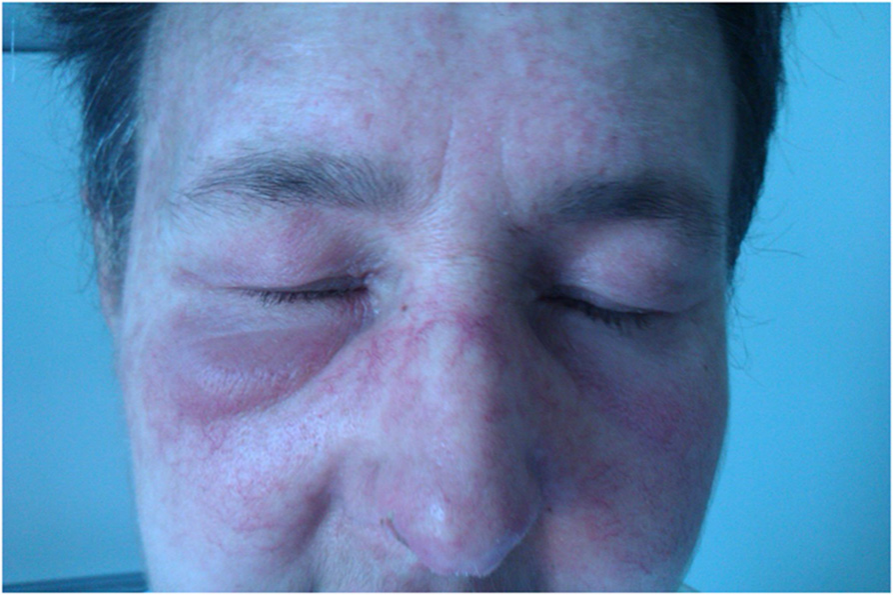
**Periorbital edema and macular exanthem of the face.**

**Figure 3:**
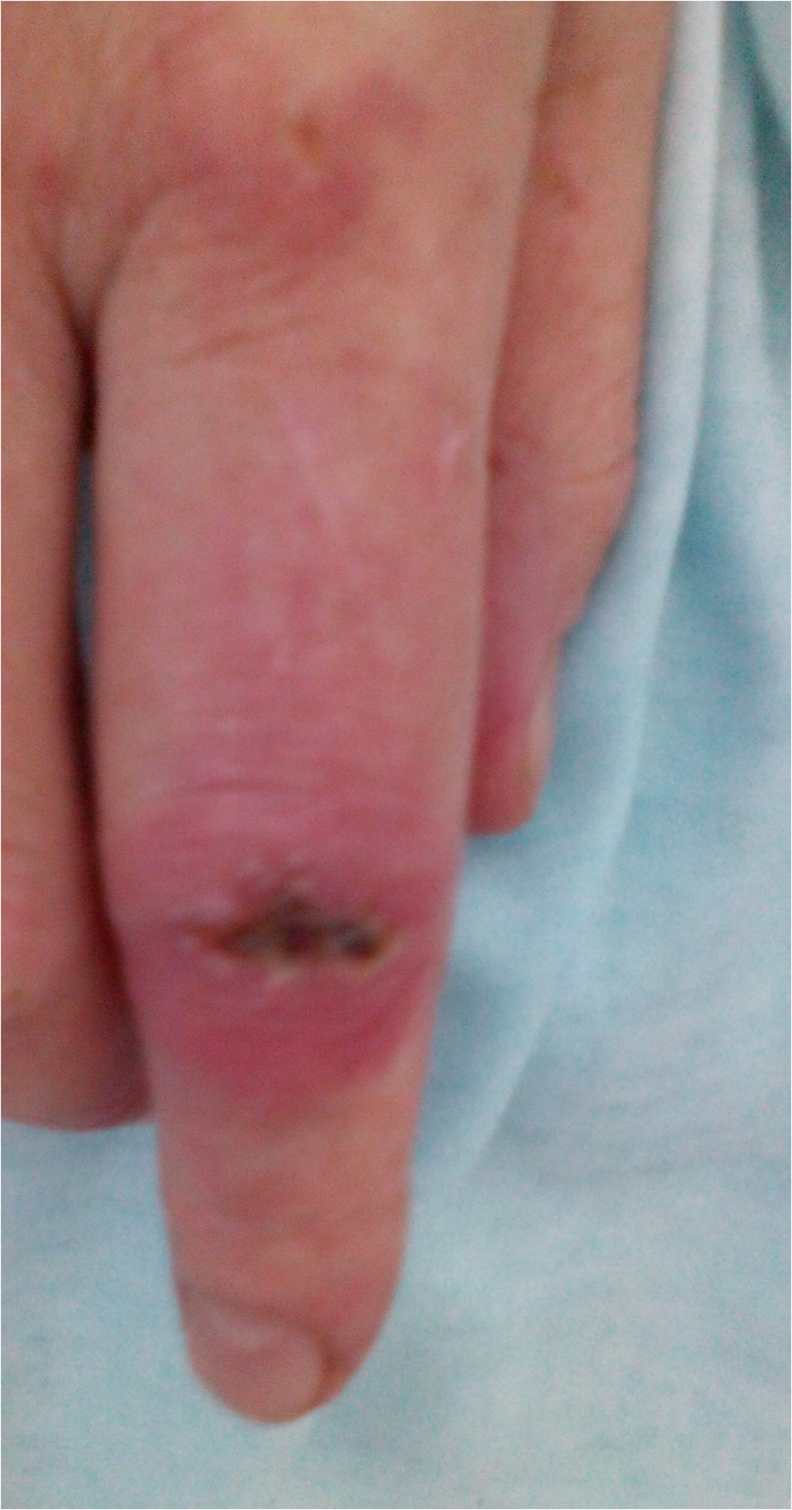
**Gottron’s papules on the metacarpophalangeal and interphalangeal joints.**

Oncologist suggested treatment with cisplatin 25 mg/m^2^ (per square meter of body-surface area) and gemcitabine 1,000 mg/m^2^, each administered on days 1 and 8 and repeated every 21 days for up to 6 cycles, unless disease progression or unacceptable toxicity occurs. Prior to starting chemotherapy in March 2014, the dermatologist increased the corticosteroid dosage (methylprednisolone 60 mg/day) due to newly manifested periorbital edema and macular exanthem in the regions of the trunk, neck and dorsum of the hand (Figures 2 and 3). The patient refused the recommended skin biopsy. Still, there was some improvement in muscular strength.

Prior to the first cycle of chemotherapy, the patient was given two doses of erythrocyte concentrate for normocytic anaemia (haemoglobin (Hgb) 95 g/l, haematocrit (Ht) 0.345 l/l, mean corpuscular volume (MCV) 82.8 fl). After 10 days of high-dose corticosteroid therapy, the skin eruptions faded. During and after the second cycle of chemotherapy, the patient complained of general weariness and fatigue.

After 3 cycles of chemotherapy, new skin changes developed on the right upper arm and both thighs, with periorbital edema more pronounced on the left side, despite the 32 mg/day maintenance dose of methylprednisolone. A follow-up MSCT of the abdomen showed a 12-mm thickening of the cholecyst wall - a regression, compared to the initial MSCT result - and increased tumour marker levels (CEA 1.6 μg/l, CA 19-9 2,525 U/ml). During the next three chemotherapy cycles, additional skin changes manifested in the armpit and on the scalp; some marker levels also increased (CA 19-9 3,259 U/ml; the gamma-glutamyl transferase (GGT) doubled). Final MSCT of the thorax, abdomen and pelvis was performed after treatment completion in August 2014, showing a progression of hepatic changes and ascites, after which the patient was referred to palliative care.

The patient presented in the case report was treated using the standard care of gall bladder carcinoma. The Helsinki Declaration was obeyed. Ethical board of Hospital Sisters of Charity gave the approval.

### Discussion

Gallbladder carcinoma is the most common and the most malignant tumour of the biliary tract. It occurs mostly between the ages of 60 and 70 and is 2 to 6 times more common in women. Histologically, 95% of gallbladder carcinoma patients have adenocarcinoma; this was also the case with my patient, as the final histopathology report of the omentum showed. Interestingly, the patient was not aware of her long-standing cholelithiasis and had no pathological condition connected to increased risk.

Gallbladder carcinoma has an extremely bleak prognosis; only 5% to 10% of patients who undergo surgery are likely to survive the following 5 years [6]. Also, one third of patients whose surgery is finished with exploration show peritoneal and liver metastases that were not visible on MSCT or MRI. In cases of suspected disseminated malignant disease, diagnostic laparoscopy is recommended [7]. My patient’s preoperative CT of the thorax, abdomen and pelvis showed no sign of liver or peritoneal metastases.

Although skin biopsy was not performed on my patient and although she did not test positive for autoantibodies usually present in patients suffering from dermatomyositis, it is my opinion that her clinical presentation is consistent with paraneoplastic dermatomyositis. The patient underwent chemotherapy with cisplatin and gemcitabine, which was in accordance with the results of the study of phase III of patients with advanced or metastatic carcinoma. Adding cisplatin to gemcitabine, that is its interaction between gemcitabine and cisplatin afforded significant progression-free survival (median, 8.4 *versus* 6.5 months; hazard ratio (HR), 0.72; 95% confidence interval (CI), 0.57 to 0.90; *p* = .003) and overall survival benefits (median, 11.7 *versus* 8.3 months; HR, 0.70; 95% CI, 0.54 to 0.89; *p* = .002). The most common side effects include decreased WBC count and fatigue [8]. My patient never exhibited decreased WBC count, which was partly due to the effects of corticosteroid therapy. Since receiving the second cycle of chemotherapy, she complained of increased general weariness and fatigue, which could be due to the chemotherapy treatment.

In January 2015, I searched PubMed for terms *gallbladder cancer*, *gallbladder carcinoma* and *dermatomyositis* and found a total of six case reports in English describing dermatomyositis in patients suffering from gallbladder carcinoma [9-13]. All of the cases featured female patients over 44 years of age, some with present risk factors (previously verified gallstones). The time interval between the appearance of dermatomyositis and malignant disease ranged from 2 weeks to 2 years. Considering the patient was initially treated in a relatively small facility and the administered treatment did not lead to improvement of her condition, it was soon suspected she had a malignant disease.

In the cases I studied, malignant disease was diagnosed within 2 to 7 months.

My patient initially had elevated CK, slightly positive ANA IIF and negative anti-Jo-1, as described in other patients. The tumour markers were initially determined to be CEA and CA 19-9. CEA was within range, whereas CA 19-9 was 24 times above normal. Elevated serum CEA and CA 19-9 levels could be suggestive of gallbladder cancer, bearing in mind that CA 19-9 has higher specificity, and CEA a higher sensitivity [14]. Although some authors describe a complete regression of skin changes following adequate treatment, in the case of my patient, the dermatomyositis and the malignant disease progressed independently of one another [13]. Following high-dose corticosteroid therapy, the skin changes faded within the same period as described in the case report of another patient; however, after 3 cycles of maintenance therapy, they progressed, although the radiology report verified the regression of the disease.

## Conclusions

Gall bladder carcinoma should be added to the list of malignancies with dermatomyositis and has to be excluded by relevant investigation in women, especially since an inexpensive, simple and easily available initial testing exists - abdomen sonography.

## Consent

Written informed consent was obtained from the patient for publication of this case report and any accompanying images. A copy of the written consent is available for review by the Editor-in-Chief of this journal.

## Abbreviations

% eos: percent eosinophils
% lymphs: percent lymphocytes
% monos: percent monocytes
% neu: percent neutrophils
ANA IIF: antinuclear antibodies verified by indirect immunofluorescence
anti-CENP-B: CENP-B specific anti-centromere autoantibodies
anti-DNA-topo 1: anti-DNA topoisomerase 1 antibody
anti-dsDNA: anti-double-strained DNA
anti-Jo-1: antibody directed against Jo-1 protein
anti-RIBO P: autoantibodies to ribosomal P
anti-Sm: anti-Sm antibodies directed against 7 proteins
anti-Sm/RNP: autoantibodies directed against the RNP/Sm ribonucleoprotein complex
anti-SS-A52: anti-Ro/SSA antibodies
anti-SS-A60: anti-Ro/SSA antibodies
anti-SSB: SSB/La antibody
CA 19-9: carbohydrate antigen 19-9
CEA: carcinoembryonic antigen
CI: confidence interval
CK: creatine kinase
DM: dermatomyositis
GGT: gamma-glutamyl transferase
Hgb: haemoglobin
HR: hazard ratio
Ht: haematocrit
L: leukocyte
MCV: mean corpuscular volume
mm: millimeter
MSCT: multi-slice computed tomography
SE: sedimentation
WBC: white blood cells
